# Pten regulates homeostasis and inflammation-induced migration of myelocytes in zebrafish

**DOI:** 10.1186/1756-8722-7-17

**Published:** 2014-03-05

**Authors:** Zhi-Wei Dong, Chun-Guang Ren, Yu Xia, Dan Su, Ting-Ting Du, Hong-Bo Fan, Hao Yuan, Lei Wang, Mei Dong, Wei-Chun Li, Yi Jin, Yi Chen, Min Deng, Ting-Xi Liu, Ai-Hua Gu, Yong Zhou

**Affiliations:** 1 Key Laboratory of Stem Cell Biology, State Key Laboratory for Medical Genomics and Laboratory of Development and Diseases, Institute of Health Sciences, Shanghai Institutes for Biological Sciences, Graduate School of the Chinese Academy of Sciences, Shanghai 200025, China; 2 Shanghai Institute of Hematology, RuiJin Hospital, Shanghai Jiao Tong University School of Medicine, Shanghai 200025, China; 3 State Key Laboratory of Reproductive Medicine, Institute of Toxicology, School of Public Health, Nanjing Medical University, Nanjing 210029, China

**Keywords:** *pten*, *cebpa*, Myeloid cells, Definitive myelopoiesis, Zebrafish

## Abstract

**Background:**

Loss of the tumor suppressor phosphatase and tensin homolog (PTEN) is frequently observed in hematopoietic malignancies. Although PTEN has been implicated in maintaining the quiescence of hematopoietic stem cells (HSCs), its role in hematopoiesis during ontogeny remains largely unexplored.

**Methods:**

The expression of hematopoietic marker genes was analyzed via whole mount *in situ* hybridization assay in *ptena* and *ptenb* double mutant zebrafish. The embryonic myelopoiesis was characterized by living imaging and whole mount *in situ* immunofluorescence with confocal microscopy, as well as cell-specific chemical staining for neutrophils and macrophages. Analyses of the involved signaling pathway were carried out by inhibitor treatment and mRNA injection.

**Results:**

*Pten*-deficient zebrafish embryos exhibited a strikingly increased number of myeloid cells, which were further characterized as being immune deficient. In accordance with this finding, the inhibition of phosphoinositide 3-kinase (PI3K) or the mechanistic target of rapamycin (mTOR) corrected the expansive myelopoiesis in the *pten*-deficient embryos. Further mechanistic studies revealed that the expression of *cebpa*, a critical transcription factor in myeloid precursor cells, was downregulated in the *pten*-deficient myeloid cells, whereas the injection of *cebpa* mRNA markedly ameliorated the dysmyelopoiesis induced by the loss of *pten*.

**Conclusions:**

Our data provide *in vivo* evidence that definitive myelopoiesis in zebrafish is critically regulated by *pten* via the elevation of *cebpa* expression.

## Background

Phosphatase and tensin homolog deleted on chromosome 10 (*PTEN)* is a well-characterized tumor suppressor gene, and *PTEN* deficiency is frequently observed in various types of cancers, including brain cancer, breast cancer, prostate cancer, endometrial carcinoma, melanoma, and leukemia. Indeed, *PTEN* is one of the most commonly mutated genes in human cancers [1-3], and germline mutations in *PTEN* are considered to be responsible for Cowden syndrome, Bannayan-Zonana syndrome, and Lhermitte-Duclose disease [4]. In addition, *PTEN* has been shown to regulate a series of fundamental cell behaviors, including cell growth, proliferation, survival, and migration, mainly by suppressing the activity of the PI3K/AKT pathway [5]*.*

Conditional ablation of *Pten* in the hematopoietic stem cells (HSCs) of adult mice leads to rapid HSC depletion and the formation of leukemia-initiating cells (LICs) [6,7]. A recent study further showed that the mechanistic target of rapamycin (mTOR) activation upon *Pten* deletion is critical during rapid HSC depletion [8]. Moreover, mTOR complex 1 (mTORC1) is also required to sustain normal hematopoiesis [9,10].

CCAAT enhancer-binding protein-α (C/EBPα) is another tumor suppressor that can inhibit cell proliferation [11,12], and mutations in *CEBPA* are widely reported in acute myeloid leukemia (AML) patients [13-16]. C/ebpα-deficient mice show a phenotype similar to AML in which the transition from the common myeloid progenitor to the granulocyte/monocyte progenitor is blocked [17,18]. In fact, C/EBPα plays a vital role in myeloid differentiation by directly enhancing the transcription of many myeloid-specific genes [19-22]. Our previous *in vitro* study showed some relation between *PTEN* and *CEBPA* in HL-60 cell line (derived from a patient with acute promyelocytic leukemia) [23], but their interactive mechanism underlying the early hematopoiesis *in vivo* still remains elusive.

Over the past two decades, the zebrafish has emerged as an ideal system for studying hematopoiesis owing to its unique advantages, including optical clarity and a high fecundity [24,25]. Similar to mammals, hematopoiesis in zebrafish consists of two successive waves: primitive and definitive hematopoiesis. Primitive hematopoiesis predominantly produces primitive erythrocytes and macrophages, whereas definitive hematopoiesis gives rise to all mature hematopoietic lineages. In zebrafish, the latter process is initiated in the ventral wall of the dorsal aorta, which is counterpart of the aorta-gonad-mesonephros (AGM) region in mammals. The hematopoietic stem/progenitor cells (HSPCs) derived from the AGM migrate to caudal hematopoietic tissue (CHT), which is similar to the fetal liver in mammals. Lastly, HSPCs colonize the kidney marrow, which is the equivalent of bone marrow in mammals, and the thymus to sustain long-term hematopoiesis throughout adulthood [25-27].

Two *pten* genes, *ptena* and *ptenb*, have been previously identified in zebrafish [28]. Although *ptena* and *ptenb* single mutants are able to survive to sexual maturity because of the overlapping functions of these genes [29]. *Ptena* and *ptenb* double mutants die approximately 5 days post fertilization (dpf) and exhibit significant hyperplastic-dysplastic phenotypes (enlarged head and heart edema. *et al.*) [29]. Accordingly, homozygous deletion of *Pten* in mice is embryonic lethal [30]. Although several elegant reports have established the pivotal role of *PTEN* in preventing leukemogenesis [6,7,10], *in vivo* evidence for *PTEN* regulation of hematopoiesis in early development is lacking, and the detailed mechanism underlying this process is still largely unknown.

In this study, we investigated the physiological role of *pten* signaling in hematopoiesis by utilizing *pten* mutant zebrafish. We tried to explore the influence of complete loss of *pten* on primitive hematopoiesis, the developmental process and the innate immune response of myeloid cells in definitive hematopoiesis, and the regulational effects of the PI3K/mTOR pathway involved. Furthermore, we revealed the intriguing function of overexpression of C/ebpα in the hematopoietic defect of *pten* mutant embryos by acting downstream of the PI3K pathway.

## Results

### Loss of *pten* induces abnormal hematopoiesis in zebrafish larvae

To evaluate the role of *pten* in hematopoiesis, we examined the expression of critical hematopoietic genes in *pten*-deficient zebrafish by using whole-mount *in situ* hybridization analysis.

We first examined hematopoiesis in *ptena* and *ptenb* single-mutant embryos and found no obvious alteration in hematopoiesis (data not shown). We then examined the hematopoietic phenotypes in *ptena* and *ptenb* double-mutant (*ptena*−/−*ptenb*−/−, hereafter referred to as *pten*−/−) embryos, which were derived from an incross of *ptena*+/−*ptenb*−/− (hereafter referred to as *pten*+/−) zebrafish. Both primitive hematopoiesis at 22 hours post-fertilization (hpf) (Additional file 1: Figure S1A-H) and definitive hematopoiesis at 36 hpf and 48 hpf (Additional file 1: Figure S1I-P) were normal in the *pten*−/− embryos.

However, in comparison to the control embryos, the *pten*−/− embryos at 90 hpf showed an obvious increase in the number of *cmyb-*expressing and *scl*-expressing HSPCs in the CHT (Additional file 1: Figure S2A-D). Of note, a significant increase in the number of *α-E1 globin*-expressing mature erythrocytes that were ectopically dispersed in the head and yolk sac of the *pten*−/− embryos was observed, although only subtle expansion was noted in the CHT (Additional file 1: Figure S2E-F), the major region colonized by mature erythrocytes in control embryos.

**Figure 1:**
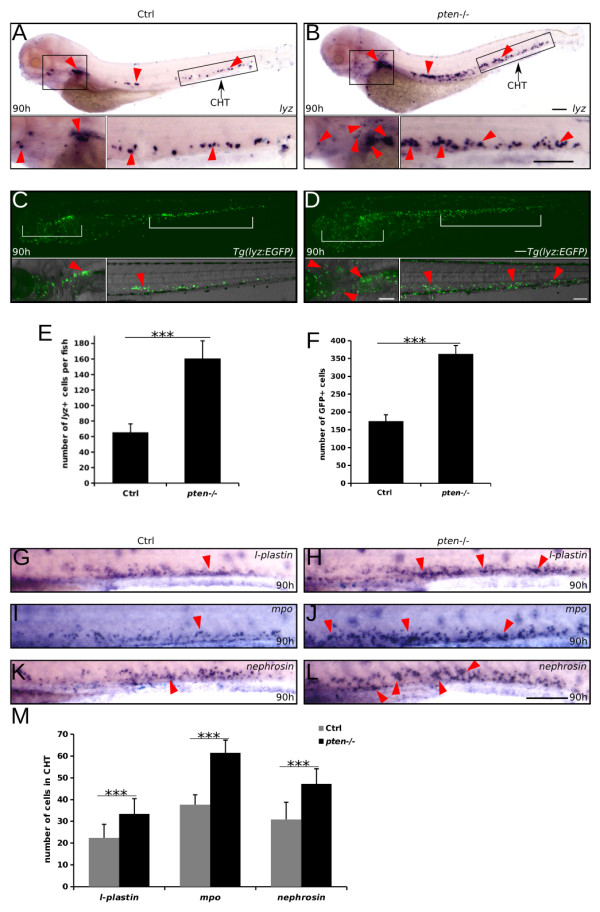
**Definitive myelopoiesis is strongly expansive in *pten-*deficient embryos. (A-B)** Whole-mount *in situ* hybridization analyses of definitive myelopoiesis with riboprobes for *lyz* in the indicated embryos at 90 hpf. The lower panels are high-magnification views of the indicated region in the upper panel. The red arrowheads indicate myeloid cells in the CHT and kidney/thymus region. **(C-D)** The *pten−/−;lyz:EGFP* embryos show increased EGFP-positive myeloid cells. The red arrowheads indicate EGFP-positive cells in the CHT and kidney region. The embryos are shown in lateral views with the anterior to the left. e: eye. **(E-F)** Statistical result for a, b and c, d respectively. **(G-M)** Whole-mount *in situ* hybridization analyses for other myeloid markers (*l-plastin, mpo, nephrosin*) in the CHT*.* The red arrowheads indicate the myeloid cells marked by each probe. The data shown in **(E-F)** and **(M)** are the means ± SEM of at least 30 embryos; ***p < 0.001 versus the control. Scale bar: 100 um.

We observed that definitive myelopoiesis in the *pten*−/− embryos was significantly increased at 90 hpf, as indicated by the enhanced expression of *lyz* (a myeloid marker) in both the CHT and kidney region (Figure 1A-B and E), and we also confirmed expansive myelopoiesis in the *pten−/−;lyz:EGFP* embryos [31] (Figure 1C-D and F)*.* Furthermore, we examined other markers of myeloid cells (*l-plastin, mpo,* and *nephrosin*) in addition to *lyz* and found that their expression levels were also increased in the CHT at 90 hpf (Figure 1G-M), indicating that *pten* signaling is essential for normal definitive myelopoiesis. To further characterize the developmental process of abnormal myelopoiesis in *pten*−/− fish, we performed whole-mount *in situ* hybridization for *lyz* every 6 hours, from 72 to 108 hpf (Additional file 1: Figure S3A-N and Q). The whole-mount *in situ* hybridization results showed that abnormal myelopoiesis appeared as early as 78 hpf (Additional file 1: Figure S3A-D) and lasted for approximately 15 hours until the myeloid cells became scattered along the upper edge of the yolk sac at approximately 96 hpf (Additional file 1: Figure S3C-J). Lastly, the myeloid cells of the *pten−/−* embryos became much more dispersed, and their number decreased to a level comparable to that of the controls (Additional file 1: Figure S3K-N), which is likely due to enhanced apoptosis (Additional file 1: Figure S3O-P and R). However, it should be noticed that apoptosis is not limited to *lyz*-positive cells (Additional file 1: Figure S3P). This may be due to the rapidly deteriorated health of *pten−/−* fish with faint heart beating, smaller eyes and enlarged head from 4 to 5 dpf. In fact, *pten−/−* embryos only had slightly visible abnormality (subtle heart edema) at 90 hpf when most of our assays were performed. Thus, the marked changes at late stage hardly impacted on our observation in myelopoiesis.

Taken together, these results demonstrate that a deficiency in *pten* signaling induces abnormal hematopoiesis, particularly the abnormal expansion of definitive myeloid cells, in zebrafish.

### Expansive myelopoiesis in *pten*−/− embryos is due to reduced apoptosis and block in myeloid cell maturation

To determine whether the relatively expansive myelopoiesis in *pten*−/− embryos can be attributed to the enhanced myeloid cell proliferation, we performed double staining in *pten−/−;lyz:EGFP* embryos using antibodies against phospho-histone 3 (PH3) and EGFP. There was no significant difference in the proliferation of *lyz*-positive myeloid cells between the *pten*−/− and control embryos (Additional file 1: Figure S4A-F and M). In addition, no obvious changes in the proliferation of HSPCs was observed in the *pten−/−* embryos, as evidenced by the co-staining of PH3 and EGFP in *pten−/−;cmyb:EGFP* larvae (Additional file 1: Figure S4G-L and N).

Notably, the number of myeloid cells in the control embryos at 90 hpf was significantly lower than that at 80 hpf; however, the myeloid cell number in the *pten*−/− embryos at 90 hpf was almost the same with that at 80 hpf (Additional file 1: Figure S3, the second and the forth value on the dashed line, both of which is about 170). We hypothesized that the expansion of myeloid cells was due to the relative reduction in apoptosis in the *pten*−/− embryos. To test this possibility, a terminal transferase UTP nick end-labeling (TUNEL) assay was performed at 80 hpf when the *lyz*-positive cells in *pten−/−* zebrafish just started to increase. Indeed, fewer apoptotic myeloid cells were observed in the *pten*−/− embryos compared to the control embryos, suggesting that reduced apoptosis in the *pten*−/− embryos may partially contribute to the observed expansive myelopoiesis.

**Figure 2:**
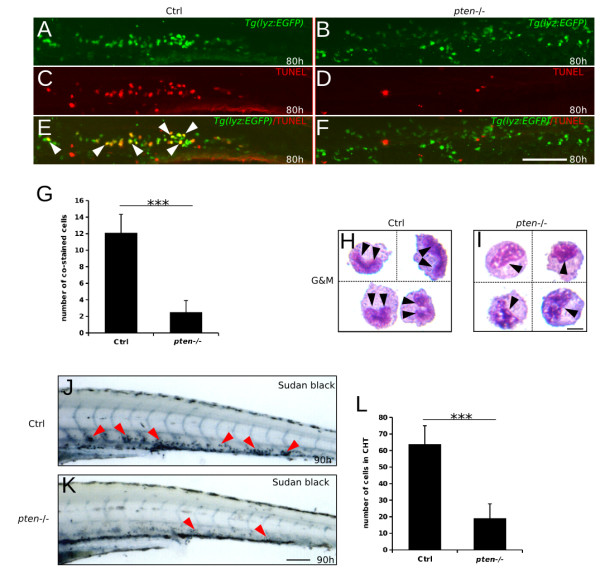
**Reduced apoptosis and block in myeloid cell maturation underlie *pten *deficiency-induced expansive myelopoiesis. (A-G)** Double staining of *lyz*-driven EGFP protein and TUNEL assay in the CHT of control and *pten−/−* embryos at 80 hpf. Although relative more EGFP-positive myeloid cells were observed in the *pten*−/− embryos (**A** and **B**, all green cells were manually counted as positive), few cells were simultaneously apoptotic, as indicated by merging with TUNEL staining (**E** and **F**, white arrowheads). **(H and I)** Wright-Giemsa staining and morphological characterization of FACS-purified EGFP-positive cells from 90 hpf *pten−/−;lyz:EGFP* and control embryos. The black arrowheads indicate the nuclear shape, which was typically kidney-like and condensed in control embryos **(H)** but remained in a loose state in the *pten*−/− embryos **(I)**. **(J-L)** Sudan Black staining of neutrophils at 90 hpf. The red arrowheads indicate neutrophils in the CHT. The data shown in **(G)** and **(L)** are the means ± SEM of at least 15 and 30 embryos; ***p < 0.001 versus the control. Scale bar: h and i, 5 um; others, 100 um.

Considering that the block in maturation could also contribute to an increased number of myeloid cells, we next examined the morphology of the myeloid cells by performing Wright-Giemsa staining of FACS-purified EGFP-positive cells from *pten−/−;lyz:EGFP* and control embryos at 90 hpf. The nuclei of the control myeloid cells exhibited a typical kidney shape, which is indicative of mature neutrophils (Figure 2H). However, the nuclei of the *pten−/−* myeloid cells exhibited a high nucleus-to-cytoplasm ratio and loose chromatin, which are features of metamyelocytes and promonocytes (Figure 2I). These data suggest that neutrophil maturation is blocked at the metamyleocytic stage in the *pten*−/− embryos.

To further confirm the reduction in mature neutrophils, we performed whole-mount *in situ* staining utilizing Sudan Black B, which specifically stains the granules of granulocytes [32,33]. The control embryos typically showed heavily stained neutrophils in the CHT, whereas only lightly stained cells were detected in the *pten*−/− embryos (Figure 2J-L). This result indicated that the maturation of neutrophils was severely blocked upon *pten* loss during definitive myelopoiesis.

Collectively, our results suggest that the observed expansive myelopoiesis in *pten*−/− embryos is due to the inhibition of myeloid maturation and a modest blockage of apoptosis.

### Loss of *pten* impairs the immune response of myeloid cells

**Figure 3:**
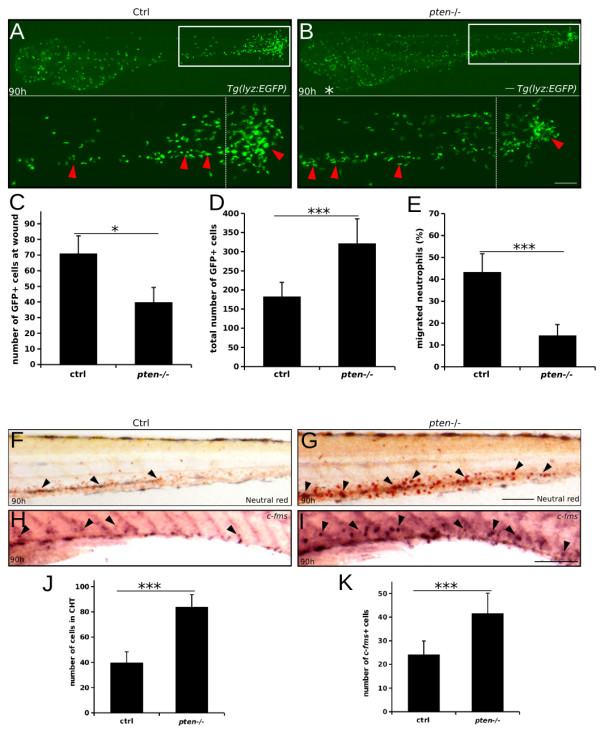
**The immune response of expansive myeloid cells is impaired in *pten*−/− embryos. (A and B)** Tail transection analyses of myeloid cell movement in response to inflammation. The myeloid cells of the control and *pten−/−* embryos moved to the wound site at 6 hours post-transection (hpt) (90 hpf) (**A** and **B**, white boxes). The red arrowheads indicate the myeloid cells away from the wound (**A** and **B**, left region of the lower panels) and the myeloid cells that arrived at the wound (**A** and **B**, right region of the lower panels). A white asterisk indicates the heart edema of *pten−/−* embryos. **(C-E)** Quantification of myeloid cells in the region to the right of the white dashed line (**A** and **B**, lower panel) **(C)** and the total number of myeloid cells **(D)** in each embryo. **(E)** The percentage of myeloid cells that migrated to the wound. **(F and G)** Neutral Red staining of macrophages at 90 hpf. The black arrowheads indicate macrophages in the CHT. **(H and I)** Whole-mount *in situ* hybridization analyses of macrophages with riboprobe for *c-fms.* The black arrowheads indicate macrophages in the CHT. **(J and K)** Statistical results of **F**, **G** and **H**, **I** respectively. The data shown are the means ± SEM of at least 30 embryos; ***p < 0.001 versus the control. Scale bar: 100 um.

Because the immune response of myeloid cells is important in the early development of zebrafish [31,34-37], we evaluated the ability of these cells to respond to inflammation in *pten*−/− embryos. Tail transections near the caudal circulatory system of *pten−/−;lyz:EGFP* and control embryos at 84 hpf were performed as previously described [31,37]. Six hours later, the directional migration of EGFP-positive cells toward the acute injury induced by the tail transections was observed by fluorescence microscopy. Compared to the control embryos, the number of EGFP-positive cells in the *pten*−/− embryos that migrated to the injury site was remarkably reduced (Figure 3A-C), particularly in the region close to the wound (Figure 3A-B, rightmost areas of the lower panels). Additionally, more EGFP-positive cells in the region far from the wound were unable to migrate to the injury site (Figure 3B, leftmost areas of the lower panels, Additional file 2: Movie S1 and Additional file 3: Movie S2). Furthermore, the increased total number of EGFP-positive myeloid cells in *pten*−/− embryos (Figure 3D) enhanced the immune-deficiency of the myeloid cells in *pten*−/− embryos, with a lower percentage of migration neutrophils (Figure 3E).

To specifically assess the immune response of macrophages in the *pten*−/− embryos, we performed Neutral Red staining by incubating zebrafish larvae in staining buffer from 80 to 90 hpf. Intriguingly, more heavily stained macrophages were observed in the CHT of *pten*−/− embryos compared to the control embryos, suggesting that the *pten*-deficient macrophages take up relatively more Neutral Red (Figure 3F-G and J). We also performed whole-mount *in situ* hybridization with *c-fms,* a specific marker for macrophages. Our result indicated that the number of macrophages was greatly increased in *pten*−/− embryos compared to controls (Figure 3H-I and K), which is consistent with our Neutral Red result. These data suggested that relatively more macrophages were produced upon *pten* loss.

Collectively, these results demonstrate that the normal immune response of myeloid cells is severely impaired during definitive myelopoiesis in *pten*−/− zebrafish embryos.

### The PI3K/mTOR pathway contributes to *pten* deficiency-induced dysmyelopoiesis

**Figure 4:**
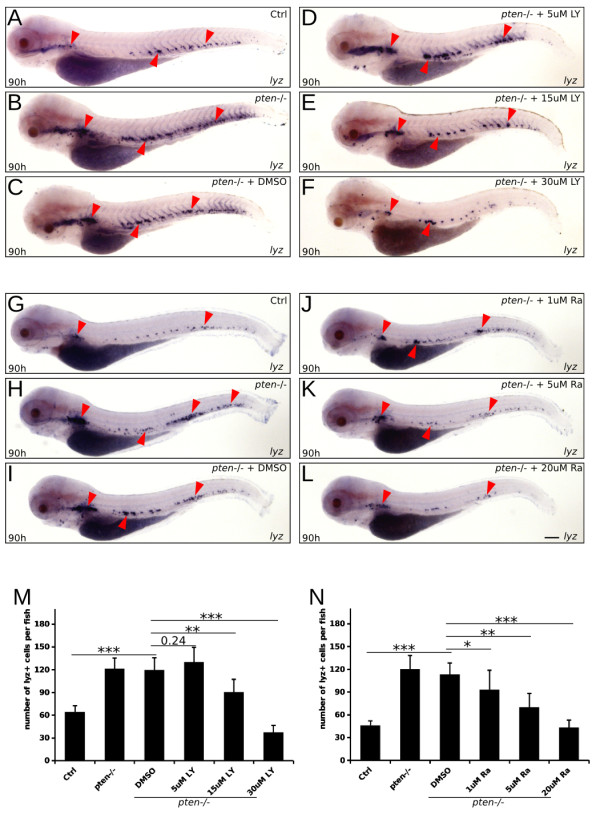
**The PI3K/mTOR pathway contributes to the expansion of definitive myelopoiesis in *pten*−/− embryos. (A-L)** Whole-mount *in situ* hybridization analyses of *lyz-*positive myeloid cells in embryos treated with increasing doses of LY294002 (LY) or rapamycin (Ra) at 90 hpf. Untreated and mock-treated *pten−/−* embryos **(B, C and H, I)** showing expansive myelopoiesis compared to the controls **(A and G)**, as mentioned above. The *pten−/−* embryos treated with stepwise increases in LY294002 or rapamycin showed an obvious rescue effect, as indicated by the decrease in myeloid cells **(D-F and I-L)**. The red arrowheads indicate the main positions of the myeloid cells. **(M and N)** Quantification of myeloid cells in each embryo (a-l). The data shown are the means ± SEM of at least 30 embryos, *p < 0.05, **p < 0.01, ***p < 0.001 versus the corresponding controls. Scale bar: 100 um.

It is well known that PTEN plays a major role in the regulation of cell survival, metabolism, and migration by negatively regulating PI3K signaling [4]. We therefore hypothesized that the expansive myelopoiesis in *pten*−/− fish may be rescued by treatment with specific inhibitors of PI3K signaling, such as LY294002. To test this hypothesis, *pten*−/− embryos were treated with LY294002 from 78 hpf to 90 hpf at concentrations of 5, 15, and 30 μM. Whole-mount *in situ* hybridization analyses showed that LY294002 rescued expansive myelopoiesis in both the CHT and kidney region in a dose-dependent manner (Figure 4A-F and M). Moreover, the *pten*−/− embryos treated with 30 μM LY294002 displayed fewer myeloid cells than the controls (Figure 4A and F).

Because mTOR signaling could be activated by the activation of the PI3K pathway [23,38,39], we also treated *pten*−/− fish with rapamycin, a specific inhibitor of mTOR. A similar dose-dependent rescue of expansive myelopoiesis was also observed (Figure 4G-L and N).

**Figure 5:**
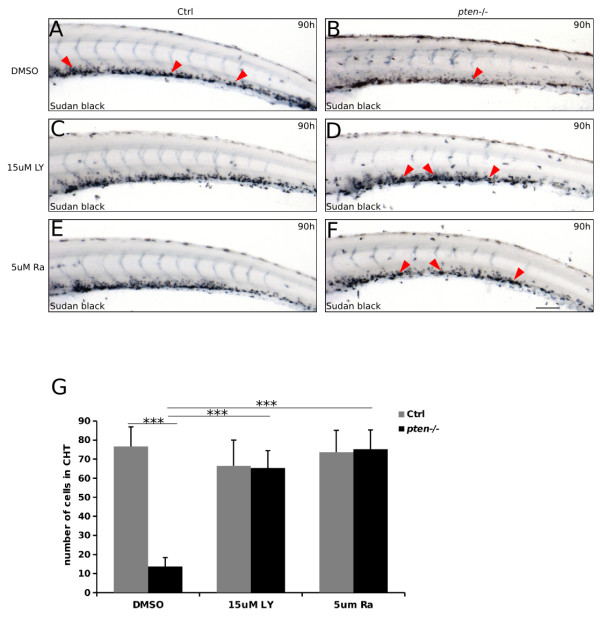
**The PI3K/mTOR pathway contributes to the blockage of myeloid cell maturation in *pten*−/− embryos. (A-F)** Sudan Black staining of neutrophils at 90 hpf. Control and *pten*−/− embryos were treated with DMSO **(A-B)** or LY294002/Rapamycin **(C-D and E-F)** at indicated concentration. The red arrowheads indicate neutrophils in the CHT. **(G)** statistical results of A-F. The data shown in **(G)** are the means ± SEM of at least 30 embryos; ***p < 0.001 versus the control. Scale bar: 100 um.

To further explore whether the suppression of PI3K/mTOR pathway could also abolish the block of neutrophil maturation upon *pten* loss, we performed Sudan black assay on LY294002- and rapamycin-treated *pten*−/− embryos (Figure 5A-F). As we speculated, the maturation of neutrophils was largely recovered in LY294002- and rapamycin-treated *pten*−/− embryos (Figure 5D and F) compared to mock-treated ones (Figure 5A and G).

We have shown that the maturation of neutrophils was blocked upon *pten* loss, which may imply that the rescue effects of LY294002 and rapamycin occurred in a cell-autonomous manner, a question need to be further addressed. To selectively antagonize PI3K/mTOR signaling in the myeloid cells of *pten*−/− embryos, we transiently expressed zebrafish *pten* (*ptena* and *ptenb*) by Tol2-mediated gene transfer using *lyz* promoter (Additional file 1: Figure S5). As predicted, *pten*−/− embryos with transiently expressed myeloid *pten* show less *lyz-*positive myeloid cells (Additional file 1: Figure S5D) compared to control embryos (Additional file 1: Figure S5C and E).

Taken together, our data indicate that the abnormal activation of the PI3K/mTOR pathway resulting from the deficiency of *pten* contributes to the expansive dysmyelopoiesis observed in *pten*−/− zebrafish embryos.

### C/ebpα plays a vital role in myelopoiesis downstream of *pten*

**Figure 6:**
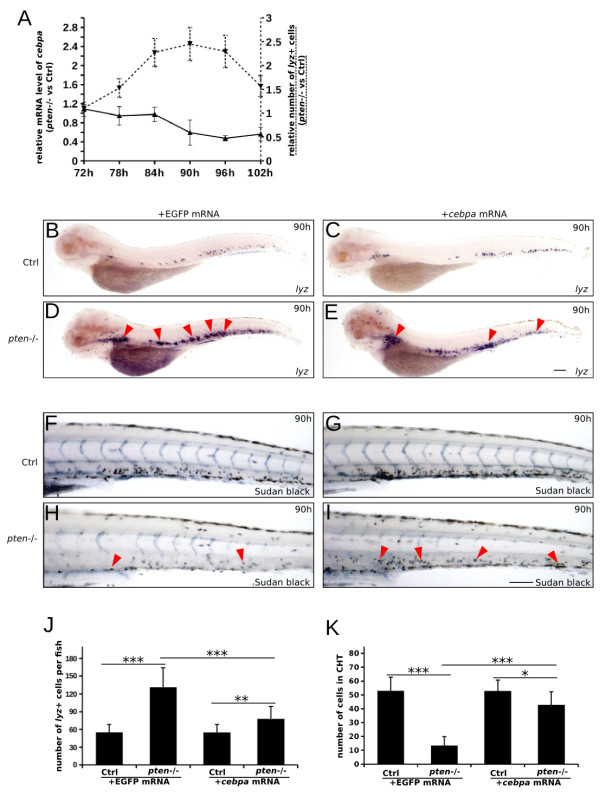
**The overexpression of C/ebpα ameliorates expansive myelopoiesis in *pten-*deficient embryos. (A)** The expression level of *cebpa* was negatively related to the number of myeloid cells. Real-time quantitative PCR of *cebpa* in EGFP-positive myeloid cells from *pten−/−;lyz:EGFP* and control embryos was performed every 6 hours from 72 to 102 hpf (solid line and left Y-axis). The *lyz-*positive myeloid cells in each embryo were counted via the whole-mount *in situ* hybridization analyses of embryos at 72 to 102 hpf (dashed line and right Y-axis). The indicated value is the ratio of *pten*−/− to control myeloid cells. **(B-E)** Whole-mount *in situ* hybridization analyses for *lyz* in embryos injected with *in vitro-*synthesized control **(B and C)** or *cebpa***(D and E)** mRNA at the one-cell stage. The overexpression of *cebpa* mRNA largely rescues expansive myelopoiesis in *pten−/−* embryos. The red arrowheads indicate expansive myelopoiesis in the mock-injected *pten*−/− embryos and the decrease in myeloid cells in the *cebpa-*injected *pten−/−* embryos. **(F-I)** Sudan Black staining of neutrophils in embryos injected with control **(F and H)** or *cebpa***(G and I)** mRNA. The red arrowheads indicate neutrophils in the CHT. **(J and K)** Quantification of myeloid cells in each embryo **(B-E and F-I)**. The data shown are the means ± SEM of at least 30 embryos; *p < 0.05, **p < 0.01, ***p < 0.001 versus the corresponding controls. Scale bar: 100 um.

Our previous study indicated that C/EBPα could regulate myeloid development downstream of *PTEN.* Elevated expression of *PTEN* could promote the expression of *CEBPA* in HL-60 cell line [23]. Moreover, C/EBPα is a well-known master regulator of myelopoiesis [40]. To further elucidate the role of C/ebpα in *pten* deficiency-induced expansive myelopoiesis, we performed real-time quantitative PCR to evaluate the expression level of *cebpa* in EGFP-positive myeloid cells isolated from *pten−/−;lyz:EGFP* embryos at every 6 hours between 72 and 102 hpf. In contrast to the gradually increasing number of *lyz*-positive myeloid cells in the *pten*−/− embryos (Additional file 1: Figure S3A-L) from 72 to 102 hpf (Figure 6A, dashed line and the right Y axes), the expression level of *cebpa* in the EGFP-positive myeloid cells gradually decreased (Figure 6A, solid line and the left Y axes).

The downregulation of *cebpa* may contribute to expansive myelopoiesis in the *pten*−/− embryos. We therefore microinjected *cebpa* mRNA into one-cell-stage *pten−/−* embryos and then performed whole-mount *in situ* hybridization analyses on the injected embryos using a probe for *lyz* at 90 hpf. Compared to the mock-injected *pten*−/− embryos (Figure 6D), the number of *lyz*-positive myeloid cells significantly decreased in the *pten*−/− embryos injected with *cebpa* mRNA (Figure 6E and J), suggesting that C/ebpα functions downstream of *pten* to regulate the homeostasis of definitive myelopoiesis.

To further determine the functional consequences of *cebpa* overexpression, we performed Sudan black staining on *cebpa* mRNA*-*injected *pten*−/− embryos (Figure 6F-I). Our result showed that the number of mature neutrophils was obviously rescued in *pten*−/− embryos injected with *cebpa* mRNA (Figure 6I), in contrast to the mock-injected ones (Figure 6H and K). These data suggested that overexpression of *cebpa* mRNA could abolish the block in neutrophil maturation induced by *pten*-dificiency.

We next examined whether the PI3K/mTOR pathway was involved in the contribution of C/ebpα to expansive myelopoiesis. To test this possibility, real-time quantitative PCR was performed to detect the expression level of *cebpa* in *lyz-*positive myeloid cells treated with LY294002 or rapamycin. Surprisingly, the elevated expression of *cebpa* was observed only in the LY294002-treated embryos (Additional file 1: Figure S6), suggesting that C/ebpα functions downstream of *pten* in a PI3K-dependent, mTOR-independent manner.

Collectively, our results demonstrate that the downregulation of *cebpa* by the PI3K pathway contributes to *pten* deficiency-induced expansive myelopoiesis.

## Discussion

More than a decade of work has been performed to examine the role of PTEN, which is a frequently mutated tumor suppressor gene [41]. In this study, we investigated the role of *pten* in the developmental process of hematopoietic lineages, which has not been fully characterized. Our data revealed an essential role for *pten* in definitive hematopoiesis, particularly myelopoiesis. Importantly, we determined that immune-deficient cells are produced during the expansive myelopoiesis caused by the loss of *pten*. Moreover, this expansive myelopoiesis could be completely rescued by treatment with inhibitors of the PI3K/mTOR pathway. Additionally, we found that the expression of *cebpa* is regulated by PTEN/PI3K signaling and that *cebpa* downregulation contributes to the expansive myelopoiesis induced by *pten* deficiency. Collectively, our results help to elucidate the previously obscure role of *pten* in zebrafish myelopoiesis and the innate immune response in addition to the critical role of C/ebpα, which functions downstream of *pten* in the regulation of myelopoiesis.

Our previous report indicated that *ptenb* morphants develop myelodysplasia during primitive hematopoiesis [23]; however, this was not observed in our *ptenb* and *pten*−/− mutants (data not shown and Additional file 1: Figure S1). This discrepancy may be due to the toxicity of the morpholino and/or cross-reactions of the *ptenb* morpholino with other unknown transcripts. Nonetheless, we did observe abnormal hematopoiesis at a later stage (90 hpf) in the *pten*−/− embryos, which further confirmed the indispensability of *pten* in hematopoiesis. The most striking phenotype that we observed in the *pten*−/− embryos was expansive myelopoiesis (Figure 1A-B and E), which was specific to the developmental stage and could not be detected at 72 hpf (Additional file 1: Figure S3A-B), as indicated by the whole-mount *in situ* hybridization staining of *lyz*. Considering that our *pten*−/− embryos were obtained by incrossing *ptena*+/−*ptenb*−/− fish, the maternal contribution of *ptena*[42] might have contributed to the developmental stage-specific occurrence of expansive myelopoiesis, which might no longer be observable, when the maternal influence of *ptena* eventually dissipates.

Despite the relative increased staining of HSPCs (Additional file 1: Figure S2A-D), we observed dispersed erythrocytes in the head region (Additional file 1: Figure S2E-F), which might have been caused by vascular and/or circulation defects [43].

Similar to mammals, zebrafish myeloid cells (mainly neutrophils and macrophages) are important partners of the innate immune system [33,36,44]. However, the exact function of *pten* in myeloid cells has remained unclear. In mice, *Pten* was first highlighted as a suppressor of myeloid cell migration [45]. Conversely, another study indicated that *Pten* functioned to promote neutrophil influx during inflammation [46]. Our results clearly show that *pten* is indispensable for the maturation of neutrophils, ensuring normal migration during the inflammation induced by acute injury (Figure 2J-L and Figure 3A-C). Interestingly, a recent study indicated that *Pten* loss could arrest differentiation, which may help to explain impaired immune function of myelocytes [47]. Moreover, our data also suggest that the means by which *pten* regulates macrophage function are largely different from the means by which it regulates neutrophils, as more Neutral Red absorption was observed in the relatively more macrophages of the *pten* deficient embryos but almost no mature neutrophils was observed in these embryos (Figure 3F-G and Figure 2J-L). Although the phagocytosis ability of *pten*−/− macrophages might be greatly elevated, which was previously indicated in *ex vivo* murine peritoneal and alveolar macrophages [48-50], other possibilities should not be excluded. For example, *pten*−/− macrophages might be less intact and in turn could easily be saturated by Neutral Red. A more critical functional assay of *pten*−/− macrophages may help to clarify this issue.

In addition to the characterization of expansive myelopoiesis and the impaired immune response of myeloid cells induced by *pten* deficiency, this study also indicated the contribution of C/ebpα and PI3K/mTOR signaling in *pten*-regulated myelopoiesis (Figures 4, 5 and 6). Indeed, the contribution of PI3K/mTOR signaling was further clarified in our *pten*-deficient zebrafish model showing expansive myelopoiesis (Figures 4 and 5), though the possibility a PI3K-independent function of *pten* was responsible for this phenotype could not be completely excluded. Although non-specificity of LY294002 at high doses was reported before [51] and this non-specificity was not easy to assess, the increasing rescue effect of dysmyelopoiesis with increasing dose of LY294002 could still support our conclusion. In fact, no additional obvious abnormality was observed in *pten−/−* fish treated with 30 μM LY294002 compared to mock treated ones. Our previous study indicated that C/EBPα may act as a downstream effector of *PTEN* in hematopoiesis [23]. Moreover, other study shown that PTEN/PI3K signaling (through regulating eIF4E and eIF2) could influence the expression of CEBPA [52]. Interestingly, our data strongly suggest that the elevated expression of *cebpa* could significantly ameliorate *pten* deficiency-induced expansive myelopoiesis (Figure 6); however, more research is still needed to determine the detailed mechanism of C/ebpα regulation via the *pten* pathway.

Our results also indicated that the regulation of C/ebpα by *pten* is PI3K dependent and mTOR independent though inhibition of the mTOR pathway could rescue the expansive myelopoiesis induced by the loss of *pten* (Additional file 1: Figure S6 and Figure 4G-L). This result is conceivable considering that the phosphorylation of Akt at Ser 473 is also regulated in a PI3K-dependent, mTOR-independent manner [53]. We propose that some unknown factors that underlie *pten* deficiency-induced expansive myelopoiesis might exist downstream of mTOR. Nevertheless, our results suggest that both mTOR-dependent and -independent mechanisms may underlie *pten*-regulated myelopoiesis; clearly, these mechanisms warrant further investigation.

## Conclusions

Our study reveals the developmental function of *pten* in myelopoiesis and further dissects its role in the immune response of myeloid cells. Our finding that C/ebpα acts as a downstream effector of *pten* provides more informative clues regarding the intrinsic nature of *pten*-regulated hematopoiesis. In addition, *pten−/−;lyz:EGFP* fish are an ideal model for performing large-scale whole-animal small molecule screening to identify drug candidates that can ameliorate defects in myelopoiesis.

## Methods

### Zebrafish maintenance and embryo production

Zebrafish maintenance, breeding, and staging were performed as described previously [54]. The zebrafish facility and zebrafish study were approved by the Institutional Review Board of the Institute of Health Sciences.

### Genomic DNA isolation and genotyping

Embryos or tail fins were incubated in lysis buffer (1 M Tris–HCl [pH 8.3], 1 M KCl, 10% Tween 20, and 10% NP40) and subsequently treated with proteinase K (10 mg/ml) at 55°C overnight. After centrifuging at 12, 000 × g for 10 minutes at 4°C, the supernatant was subjected to genomic PCR using primers specific for the indicated genes.

### Riboprobe synthesis and whole-mount mRNA *in situ* hybridization

Digoxigenin-labeled antisense RNA probes were transcribed from linearized constructs using T3, T7, or SP6 polymerase (Roche Applied Science, Indianapolis, IN, USA). Whole-mount mRNA *in situ* hybridization was performed as described previously [55]. The probes were detected using an alkaline phosphatase-coupled anti-digoxigenin Fab fragment antibody (Roche Diagnostics, Indianapolis, IN, USA) with 5-bromo-4-chloro-3-indolyl phosphate/nitro blue tetrazolium (BCIP/NBT) staining (Vector Laboratories, Burlingame, CA).

### Phosphorylated histone H3 labeling and TUNEL assay

TUNEL assays were performed using the *In Situ* Cell Death Detection Kit and TMR Red (Roche Diagnostics) according to the manufacturer’s recommendations. PH3 labeling of fixed embryos was performed by overnight incubation with a rabbit anti-phosphohistone H3 antibody (Santa Cruz Biotechnology, Santa Cruz, CA, USA) at 4°C, followed by incubation with an Alexa Fluor 488 donkey anti-rabbit secondary antibody (Invitrogen, Carlsbad, CA, USA).

### Flow cytometry analysis and cytology

*lyz:EGFP* and *cmyb:EGFP* transgenic embryos were dissected and digested with 0.5% trypsin (GIBCO, Grand Island, NY, USA) for 30 minutes at 37°C. A single-cell suspension was obtained by centrifugation at 400 × g for 5 minutes, washing twice with PBS, and passing through a 40-μM nylon mesh filter. Fluorescence-activated cell sorting (FACS) was performed using the MoFlo system (DakoCytomation, Carpinteria, CA, USA) to obtain homogenous EGFP + cells, which were subsequently subjected to cytospinning at 40 × g for 5 minutes, followed by Wright-Giemsa staining. The staining was performed according to the manufacturer’s instructions (Sigma-Aldrich, UK). The micrographs were obtained using a microscope (Nikon ECLIPSE 80i) with a 100× oil immersion objective and Nikon ACT-1 software (Nikon Corporation, JP).

### Neutral Red and Sudan Black staining

For Neutral Red staining, embryos were collected and incubated in egg water with 2.5 μg/mL Neutral Red (Sigma-Aldrich) from 80 to 90 hpf [56]. For Sudan Black staining, fixed embryos were treated with a Sudan Black (Sigma-Aldrich) solution, as previously described [32]. Staining was then observed under a microscope.

### Treatment of embryos with small molecule inhibitors

Small molecule inhibitors dissolved in dimethyl sulfoxide (DMSO) were added to egg water as previously described [29,57]. The concentrations of LY294002 and rapamycin were 5–30 μM and 1–20 μM, respectively. Both inhibitors were added at 78 hpf.

### mRNA synthesis and microinjection

mRNA was synthesized using the mMessage mMachine kit (Ambion, Austin, TX, USA). For the microinjection experiment, a volume of 1 nL (30 ng/μL) was injected into embryos at the one-cell stage.

### Tol2-mediated gene transfer

The zebrafish *lyz* promoter was cloned into the *EGFP-2A-pDestTol2* plasmid at the XhoI and ClaI sites to get the *lyz-pDestTol2* construct. The zebrafish *ptena* and *ptenb* were obtained by PCR from the cDNA of wild-type embryos respectively, and then utilizing overlapping PCR to get *EGFP-2A-ptena/ptenb* fragments which were cloned into the *lyz-pDestTol2* construct at the ClaI and SalI sites to get the *lyz-EGFP-2A-ptena/ptenb-pDestTol2* constructs. Then the *lyz-EGFP-2A-pDestTol2* and *lyz-EGFP-2A-ptena/ptenb-pDestTol2* transgenic plasmids (25 ng/μL) were individually co-injected with Tol2 transposase mRNA (25 ng/μL) as previously reported [58] into the *pten*−/− or control embryos at one cell stage.

### Real-time quantitative PCR

Total RNA was extracted from at least 5,000 FACS-sorted cells using the Trizol reagent (Sigma-Aldrich). RNA was reverse-transcribed using oligo (dT) and Superscript™ III reverse transcriptase (RT) (Invitrogen). The subsequent PCR assay was performed with the SYBR Premix ExTaq kit (Takara, JP) (sequence data are available upon request). The relative expression values were normalized to the internal control (*β-actin*). The cDNA amplification was performed using an ABI Prism 7900 HT cycler (Applied Biosystems, Foster City, CA, USA). Primers for *cebpa,* 5′-CAAGCAAGAGAAGCTCAAAC, 5′-ACCGTGGTGGTAGTCGTAG; for *β-actin,* 5′-TGCTGTTTTCCCCTCCATTG, 5′-TTCTGTCCCATGCCAACCA.

### Statistical analysis

The quantitative data are expressed as the mean ± SEM. The statistical significance was determined by a two-tailed Student’s t-test (*p < 0.05, **p < 0.01, ***p < 0.001).

## Competing interests

The authors declare that they have no competing financial interests.

## Authors’ contributions

ZD performed experiments and analyzed the data. CR, YX, DS, TD, HF, HY, LW, MD, WL, YJ, and YC assisted with the experiments. ZD, MD, TL, AG and YZ designed the research plan. ZD and YZ wrote the paper. All authors read and approved the final manuscript.

## Supplementary Material

Additional file 1: Figure S1 Primitive hematopoiesis and the early stage of definitive hematopoiesis are normal in *pten*-/- embryos. **Figure S2.** Definitive hematopoiesis is hampered in *pten*-/- embryos. **Figure S3**. WISH analyses of *lyz-*positive myeloid cells from 72 to 108 hpf. **Figure S4**. Dysmyelopoiesis induced by Pten loss is not due to the proliferation of hematopoietic cells. **Figure S5**. Transiently expressed Pten in myeloid cells ameliorates expansive myelopoiesis in *pten*-/- fish. **Figure S6**. The expression level of *cebpa* is regulated by PI3K rather than the mTOR pathway. Click here for file

Additional file 2: Movie S1 Movies were taken at about 88 hpf (4 hours after tail transection) and lasted for half an hour. Wound was at the right side of movies. Compared to control *lyz:EGFP* embryos. Click here for file

Additional file 3: Movie S2 Movies were taken at about 88 hpf (4 hours after tail transection) and lasted for half an hour. Wound was at the right side of movies. Most of the EGFP-positive myeloid cells in *pten*-/- embryos showed little migration to the wound. Click here for file
