# Supplementary material for: Pten regulates homeostasis and inflammation-induced migration of myelocytes in zebrafish
Source: J Hematol Oncol. 2014 Mar 5;7:17. doi: 10.1186/1756-8722-7-17 (PMC4015859; doi:10.1186/1756-8722-7-17)
Supplement: Additional file 1: Figure S1 — Primitive hematopoiesis and the early stage of definitive hematopoiesis are normal in pten-/- embryos. Figure S2. Definitive hematopoiesis is hampered in pten-/- embryos. Figure S3. WISH analyses of lyz-positive myeloid cells from 72 to 108 hpf. Figure S4. Dysmyelopoiesis induced by Pten loss is not due to the proliferation of hematopoietic cells. Figure S5. Transiently expressed Pten in myeloid cells ameliorates expansive myelopoiesis in pten-/- fish. Figure S6. The expression level of cebpa is regulated by PI3K rather than the mTOR pathway. [file 1756-8722-7-17-S1.pdf]

Figure S1.

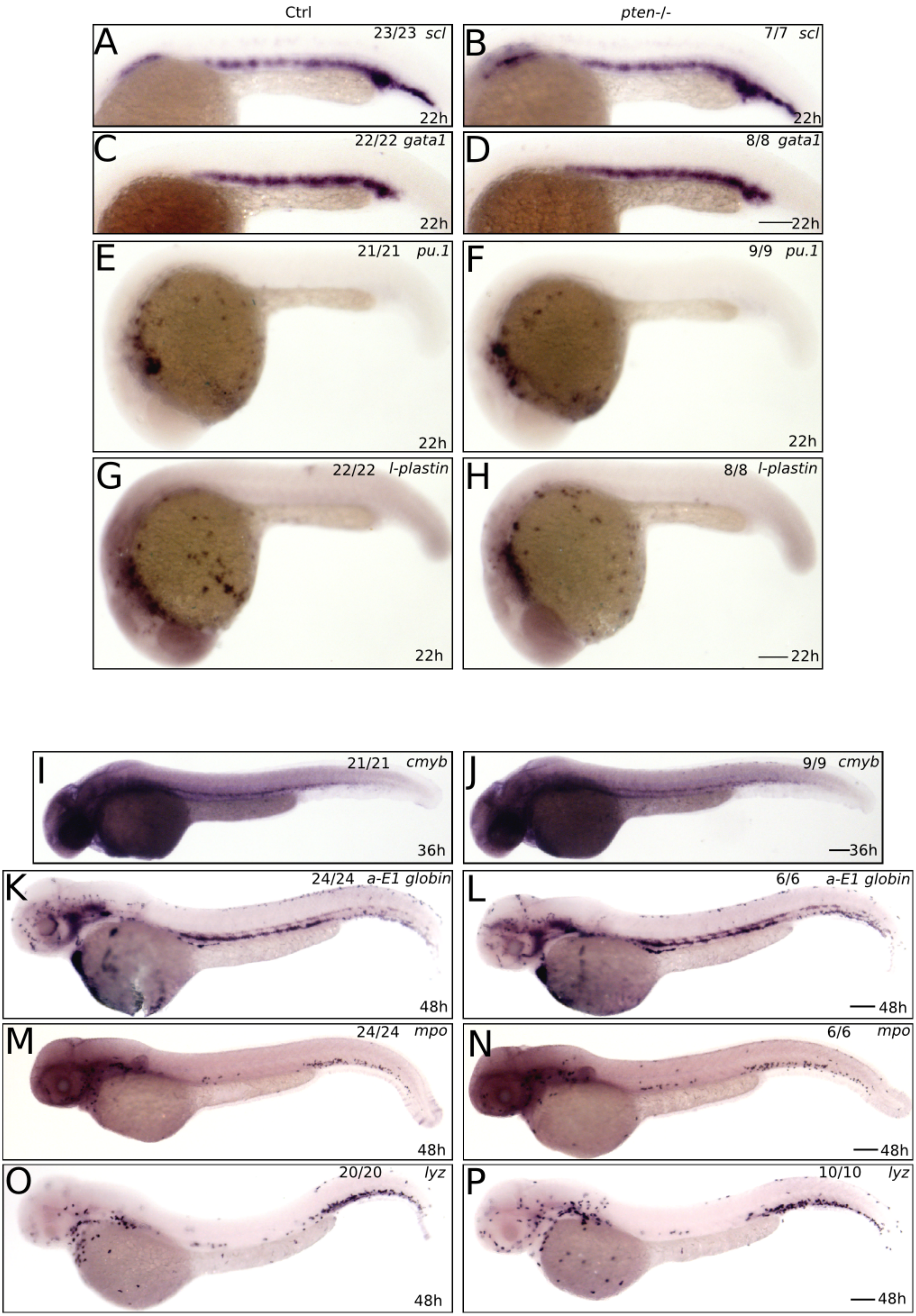
Ctrl
pten-/-
A 23/23 scl 22h
B 7/7 scl 22h
C 22/22 gata1 22h
D 8/8 gata1 22h
E 21/21 pu.1 22h
F 9/9 pu.1 22h
G 22/22 l-plastin 22h
H 8/8 l-plastin 22h
I 21/21 cmyb 36h
J 9/9 cmyb 36h
K 24/24 a-E1 globin 48h
L 6/6 a-E1 globin 48h
M 24/24 mpo 48h
N 6/6 mpo 48h
O 20/20 lyz 48h
P 10/10 lyz 48h

Figure S2.

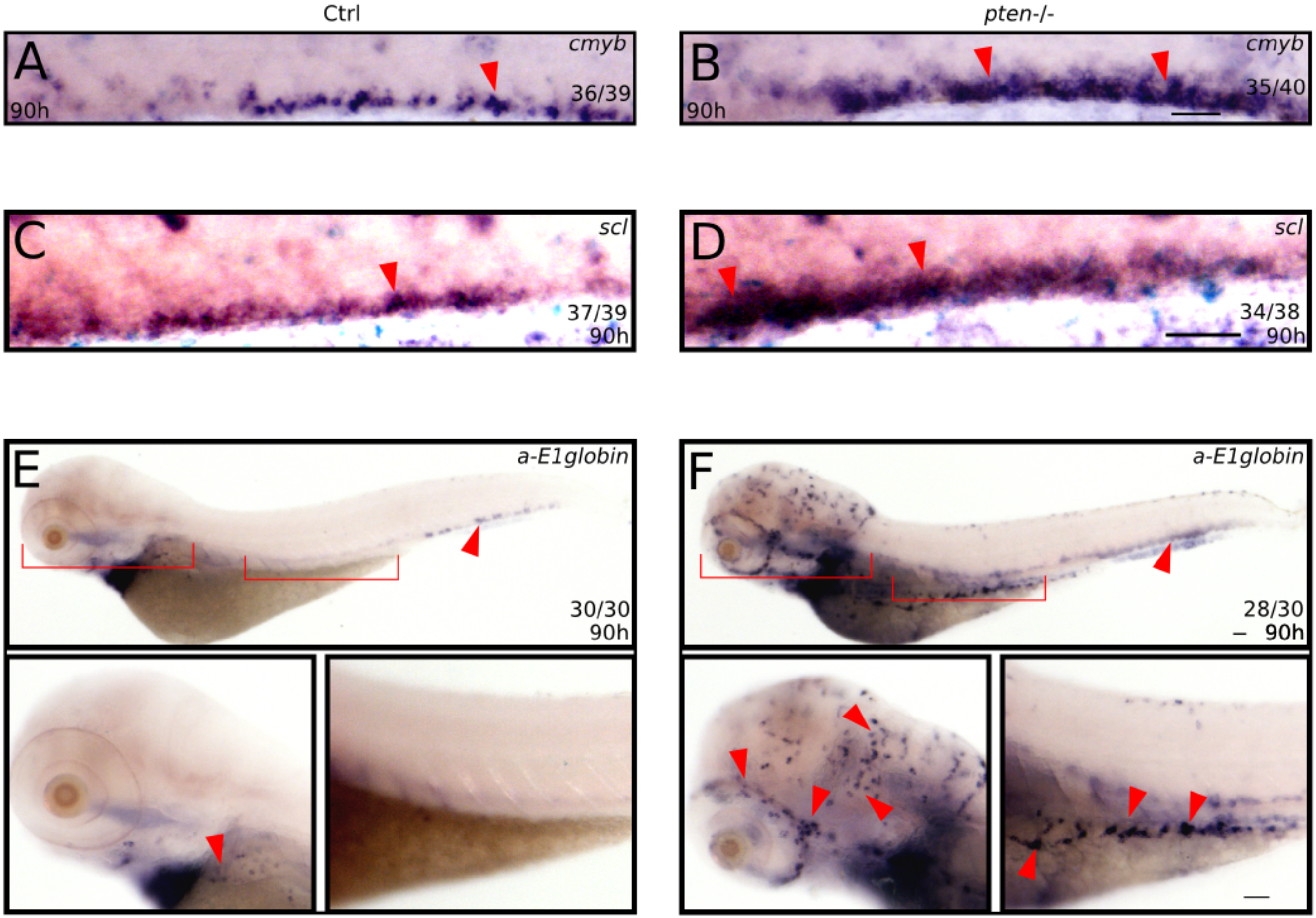

Ctrl
pten-/-
A
cmyb
36/39
90h
B
cmyb
35/40
90h
C
scl
37/39
90h
D
scl
34/38
90h
E
a-E1globin
30/30
90h
F
a-E1globin
28/30
90h

Figure S3.

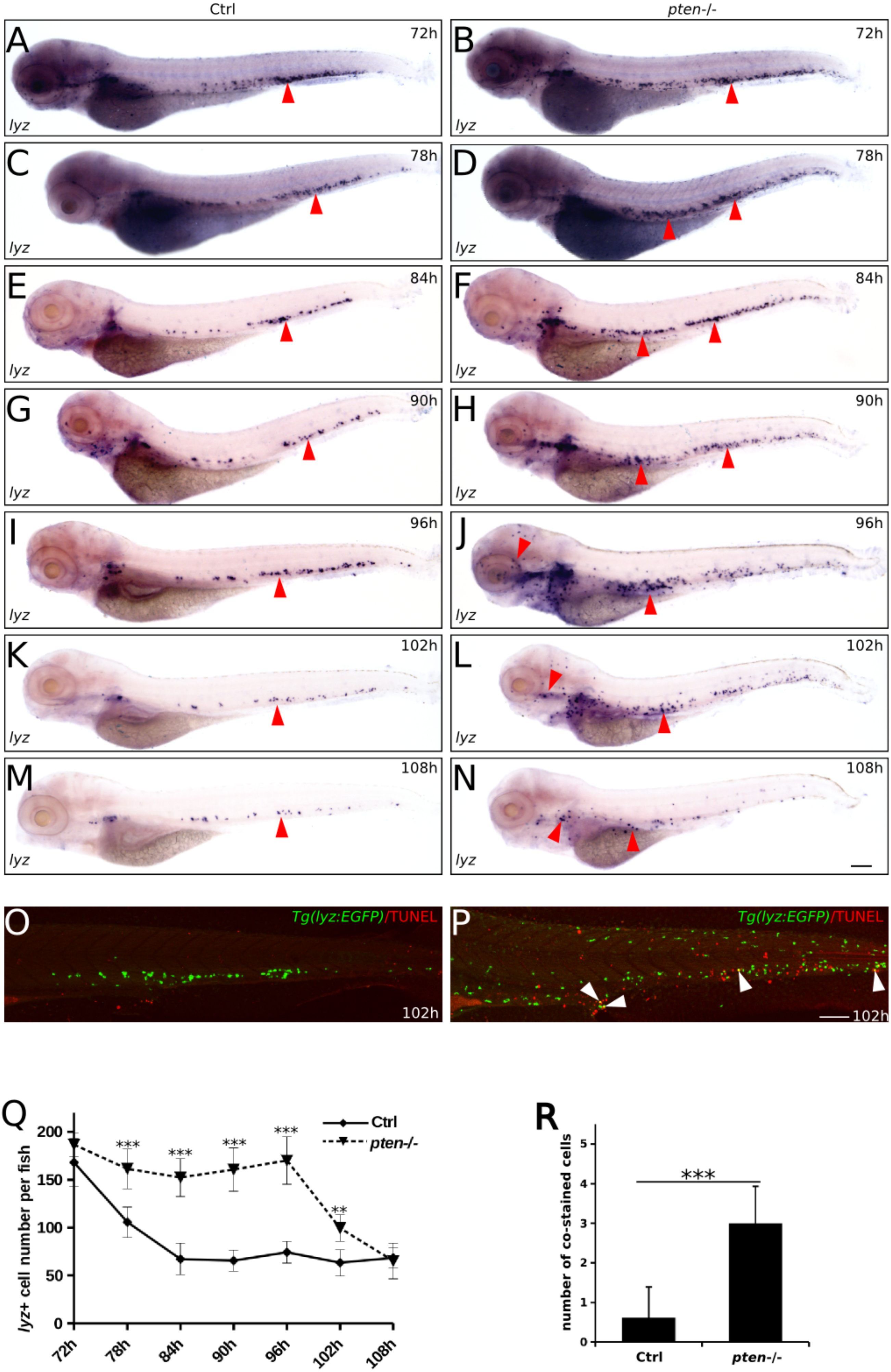

Ctrl
pten-/-
A
B
C
D
E
F
G
H
I
J
K
L
M
N
72h
78h
84h
90h
96h
102h
108h
lyz
O
P
Tg(lyz:EGFP)/TUNEL
102h
Q
Ctrl
pten-/-
lyz+ cell number per fish
***
**
200
150
100
50
0
72h
78h
84h
90h
96h
102h
108h
R
number of co-stained cells
5
4
3
2
1
0
Ctrl
pten-/-

Figure S4.

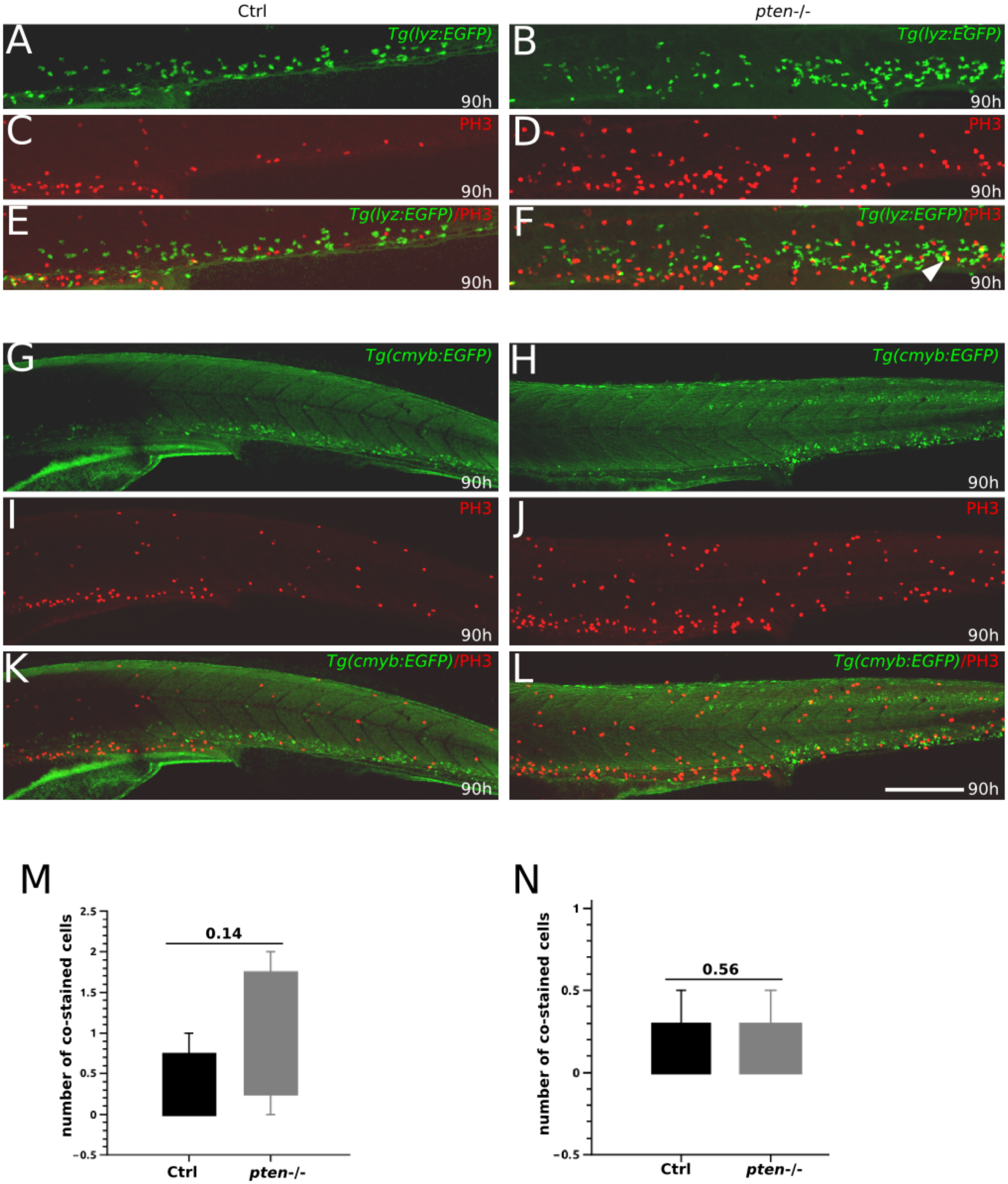

Ctrl
pten-/-
A
Tg(lyz:EGFP)
90h
B
Tg(lyz:EGFP)
90h
C
PH3
90h
D
PH3
90h
E
Tg(lyz:EGFP)/PH3
90h
F
Tg(lyz:EGFP)/PH3
90h
G
Tg(cmyb:EGFP)
90h
H
Tg(cmyb:EGFP)
90h
I
PH3
90h
J
PH3
90h
K
Tg(cmyb:EGFP)/PH3
90h
L
Tg(cmyb:EGFP)/PH3
90h
M
number of co-stained cells
2.5
2
1.5
1
0.5
0
-0.5
0.14
Ctrl
pten-/-
N
number of co-stained cells
1
0.5
0
-0.5
0.56
Ctrl
pten-/-

Figure S5.

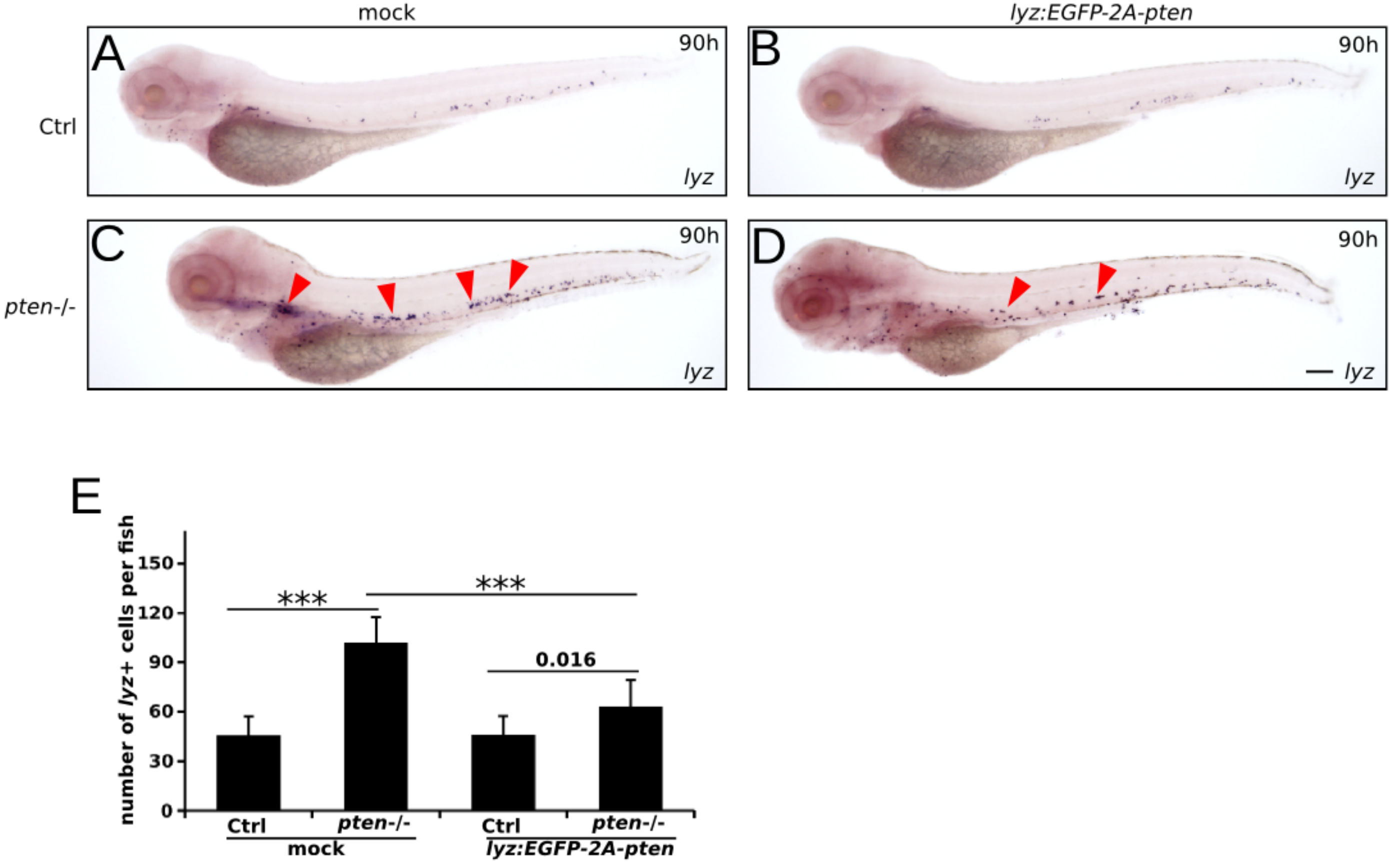
mock
lyz:EGFP-2A-pten
A
B
C
D
E
Ctrl
pten-/-
90h
lyz
number of lyz+ cells per fish
150
120
90
60
30
0
***
***
0.016
Ctrl
pten-/-
Ctrl
pten-/-
mock
lyz:EGFP-2A-pten

Figure S6.

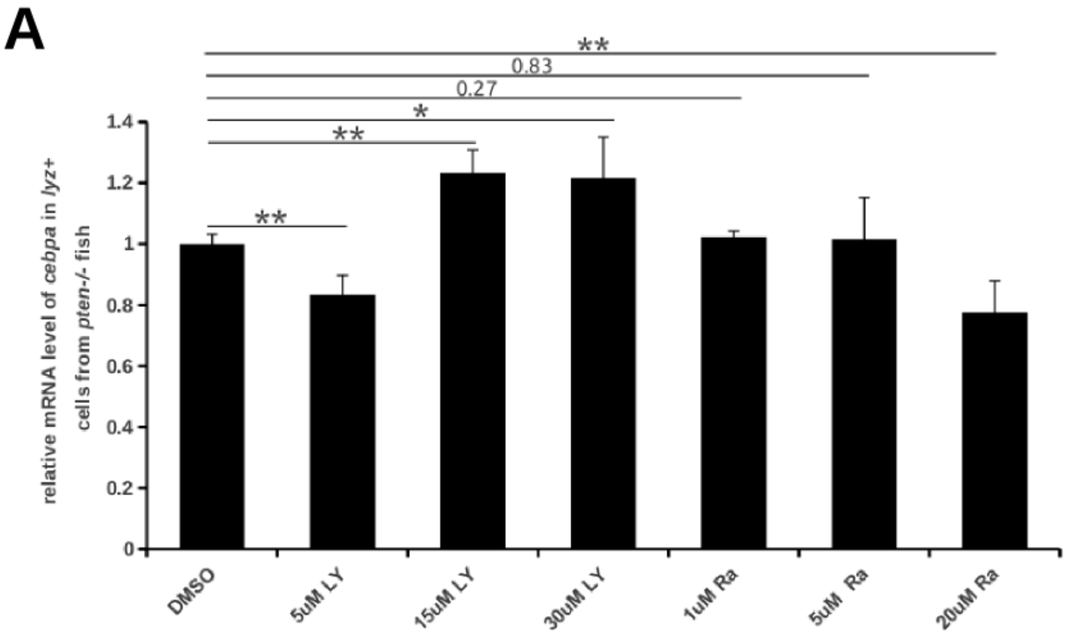
A
**
0.83
0.27
*
**
**
1.4
1.2
1
0.8
0.6
0.4
0.2
0
relative mRNA level of cebpa in lyz+ cells from pten-/- fish
DMSO
5uM LY
15uM LY
30uM LY
1uM Ra
5uM Ra
20uM Ra

Figure S1.

Primitive hematopoiesis and the early stage of definitive hematopoiesis are normal in *pten*-/- embryos.

WISH analyses of the primitive hematopoietic process at 22 hpf (A-H) and the early stage of definitive hematopoiesis at 36 hpf (I and J) and 48 hpf (K-P) in control and *pten*-/- embryos with the indicated probes (*scl* for early hematopoietic precursor cells; *gata1* for erythroid precursor cells; *pu.1* for myeloid precursor cells; *l-plastin* for early myelocytes; *cmyb* for hematopoietic precursor cells; *a-E1 globin* for mature erythrocytes; and *mpo* and *lyz* for myelocytes). Scale bar: 100um.

Figure S2.

Definitive hematopoiesis is hampered in *pten*-/- embryos.

(A-F) WISH analyses of the definitive hematopoietic process in *pten*-/- and control embryos at 90 hpf. Both *cmyb* and *scl* staining were enhanced in *pten*-/- embryos compared to the control embryos (A-D, red arrowheads). The erythrocytes marked by *a-E1 globin* exhibited an abnormal distribution, particularly in the head region and yolk sac, but exhibited normal staining in the CHT (E and F, red arrowheads). Scale bar: 50um.

Figure S3.

WISH analyses of *lyz*-positive myeloid cells from 72 to 108 hpf.

(A-N, Q) Detailed analyses of definitive myelopoiesis every 6 hours from 72 to 108 hpf. At 72 hpf, no obvious phenotype was observed in the *pten*-/- embryos (A and B, red arrowheads). Myeloid cells progressively increased beginning at 78 hpf (C-H) before becoming dispersed at 96 hpf and finally decreasing (I-N, Q).

(O-P, R) Double staining of *lyz*-driven EGFP protein and TUNEL assay in the CHT of control and *pten*-/- embryos at 102 hpf.

The data shown in (Q) and (R) are the means ± SEM of at least 20 and 15 embryos respectively; *$p < 0.05$, **$p < 0.01$, ***$p < 0.001$. Scale bar: 100um.

Figure S4.

Dysmyelopoiesis induced by Pten loss is not due to the proliferation of hematopoietic cells.

Double staining of EGFP and PH3 protein in the CHT of *pten-/-* and control embryos derived from *pten+/-;lyz:EGFP* (A-F) and *pten+/-;cmyb:EGFP* (G-L) fish at 90 hpf. The white arrowheads indicated a single myeloid cell undergoing proliferation (F). The proliferation states of myeloid cells (F) and HSPCs (L) in *pten*-/- embryos were similar to those of the control embryos (E and K, M and N).

The data shown are the means ± SEM of at least 15 embryos. Scale bar: 100um.

Figure S5.

Transiently expressed Pten in myeloid cells ameliorates expansive myelopoiesis in *pten*-/- fish.

Zebrafish Pten was transiently and selectively expressed in myeloid cells by Tol2-mediated gene transfer using *lyz* promoter. (A-E) WISH analyses of myeloid cells was performed on *lyz:pten* injected and mock-injected embryos at 90 hpf. *pten*-/- embryos injected with *lyz:pten* showed less *lyz*-positive myeloid cells (D) compred to mock-injected ones (C and E). The red arrowheads indicated *lyz*-positive myeloid cells. The data shown are the means ± SEM of at least 10 embryos; ***p < 0.001. Scale bar: 100um.

Figure S6.

The expression level of *cebpa* is regulated by PI3K rather than the mTOR pathway.

Real-time quantitative PCR analyses of *cebpa* expression in EGFP-positive myeloid cells of *pten-/-;lyz:EGFP* embryos treated with the indicated drugs. A stepwise increase in the dose of LY294002, but not rapamycin, elevated the expression of *cebpa* in the EGFP-positive myeloid cells. The data shown are the means ± SEM; *p < 0.05, **p < 0.01

versus the corresponding controls.
